# Assessment of dietary supplementation of green iron oxide nanoparticles: impact on growth performance, ammonia emissions, carcass criteria, tissue iron content, and meat quality in broiler chickens under hot climate conditions

**DOI:** 10.3389/fvets.2024.1393335

**Published:** 2024-07-02

**Authors:** Yousri A. R. Almeldin, Amira E. Eldlebshany, Enass Abd Elkhalek, Jayant Lohakare, Ahmed A. A. Abdel-Wareth

**Affiliations:** ^1^Poultry Science Department, Faculty of Agriculture, Alexandria University, Alexandria, Egypt; ^2^Poultry Center, Cooperative Agricultural Research Center, Prairie View A&M University, Prairie View, TX, United States; ^3^Department of Animal and Poultry Production, Faculty of Agriculture, South Valley University, Qena, Egypt

**Keywords:** ammonia, broilers, health, iron, nanotechnology, meat quality

## Abstract

**Background:**

The potential significance and importance of green iron nanoparticles (Nano-Fe) in poultry production lie in their capability to effectively tackle iron deficiency in poultry. Iron, an indispensable mineral for numerous physiological functions in birds, such as oxygen transport, energy metabolism, and immune response, underscores the critical need for adequate iron levels. Nevertheless, conventional iron supplementation methods frequently face hurdles like limited bioavailability rates in poultry. To enhance performance, and promote sustainable broiler productivity, Nano-Fe showed promise as an efficient feed supplement for broiler chickens. The objective of this study was to assess the impact of green Nano-Fe inclusions in diets on growth, ammonia excretion, carcass criteria, and meat quality in broiler chickens.

**Methods:**

A total of 192 one-day-old male Ross 308 broiler chicks, were assigned to three treatment diets including Nano-Fe oxide at 0, 20, or 40 mg/kg, respectively, for 42 days. Each treatment comprised eight replicates, each with eight broiler chicks. Two phases comprised the 42-day study (0 to 21 days for the starter and 21 to 42 days for the finisher).

**Results:**

In comparison to the control group, the Nano-Fe oxide groups 20 mg/kg and 40 mg/kg linearly improved (*p* < 0.05) body weight (*R*^2^ = 0.574) and body weight gain (*R*^2^ = 0.367) under hot climatic conditions at 42 days of age. Furthermore, Nano-Fe oxide to broiler diets, improved (linear, *p* < 0.05) feed conversion ratio (*R*^2^ = 0.424) throughout whole periods. The feed intake did not show any significant difference (*p* > 0.05) among groups during the experimental periods under hot climatic conditions. The ammonia content of excreta (*R*^2^ = 0.454) was linearly decreased (*p* < 0.05) with increasing Nano-Fe oxide levels in broiler diets compared to control at 21 and 42 days of age under hot climatic conditions. Nano-Fe oxide positively influences cook loss, water-holding capacity, and iron content in various tissues. Moreover, it contributes to a healthier carcass yield and reduced abdominal fat.

**Conclusion:**

In conclusion, broiler chickens fed diets containing Nano-Fe oxide at 20 mg/kg and 40 mg/kg demonstrated enhanced growth performance, improved meat quality, increased iron content in tissues, higher dressing percentage, and reduced abdominal fat deposition. Future research should explore the impact of green Nano-Fe oxide on additional factors such as the microbiome and gene expression related to immunity and heat stress.

## 1 Introduction

Iron (Fe) is essential for various physiological functions in chickens, including oxygen transport, enzyme activation, and immune system function (1). In broiler chickens, iron is primarily found in hemoglobin (60–70%), myoglobin, cytochromes, and other iron-containing enzymes (10%), alongside ferritin and hemosiderin (20–30%) (2). Maintaining systemic iron balance in poultry primarily involves controlling dietary iron uptake and release from recycling macrophages and hepatocytes, as the body does not regulate iron losses (3). However, conventional methods of iron supplementation often rely on inorganic iron sources, which can exhibit lower bioavailability and may contribute to environmental pollution through runoff and leaching (4). Moreover, Fe in broiler chicken feed can often be combined with phytate, a compound commonly found in plant-based feed ingredients like grains (5). Phytate binds to minerals such as iron, forming complexes that are not easily absorbed by the bird’s digestive system. This reduces the bioavailability of iron, meaning that even though it’s present in the feed, the chickens may not be able to absorb and utilize it effectively (6). This can lead to suboptimal iron levels in the birds, impacting their growth, health, and productivity. To address this issue, feed formulators may explore alternative forms of iron supplementation, such as Nano-Fe, which could potentially improve iron absorption and utilization in broiler chickens. Nano-Fe, or nanoscale iron particles, offers a promising solution to this problem by providing a more efficient and sustainable alternative for iron supplementation in broiler diets (7). Nano-Fe exhibits enhanced bioavailability due to its smaller particle size and increased surface area. This enhanced bioavailability allows for better absorption and utilization of iron by broiler chickens, leading to improved growth rates, feed efficiency, and overall health. Moreover, Nano-Fe supplementation has the potential to reduce the environmental burden associated with iron supplementation in broiler diets (8). By utilizing nanotechnology, Nano-Fe can be precisely engineered to deliver iron in a targeted and controlled manner, minimizing waste and reducing the need for excess supplementation (9) which may help preserve ecosystems and promotes environmental sustainability in broiler production systems. Additionally, Nano-Fe particles can be designed to be more stable in the gastrointestinal tract, ensuring that a higher proportion of the supplemented iron is absorbed by the bird rather than excreted (10). Nano-Fe offers a promising avenue for supporting sustainable broiler chicken meat production by reducing the need for dietary iron extraction (11, 12).

Green nanotechnology refers to the use of environmentally friendly processes and materials in the synthesis of nanoparticles. This approach often involves using natural sources, such as plant extracts or microorganisms, to reduce and stabilize nanoparticles. The use of green nanotechnology is driven by the desire to minimize the environmental impact and potential toxicity associated with traditional nanoparticle synthesis methods (13). Basil (*Ocimum basilicum*) in the diets of broiler chickens increased immune systems, nutrient digestibility, antioxidant activities, and productivity (14), which is mainly due to various phytochemicals, including polyphenols, flavonoids, and terpenoids (15), and may also act as stabilizing agents in the synthesis of nanoparticles.

Overall, green Nano-Fe represents may a promising solution to the problem of iron supplementation in broiler diets. Its enhanced bioavailability, targeted delivery, and reduced environmental impact make it an attractive option for poultry producers seeking to optimize broiler performance while promoting sustainability. In our prior investigation, it was demonstrated that incorporating green Nano-Fe into broiler diets, either with *Halimeda opuntia* as a carrier or independently, has the potential to enhance broiler performance and health, particularly in hot environmental conditions (16). There is still much to learn about the long-term effects, optimal dosage, and specific mechanisms of action of Nano-Fe in poultry. Continued research is necessary to fill these gaps and provide a more comprehensive understanding of their potential benefits and risks. Likewise, optimizing growth performance, improving meat quality, increasing iron content in tissues, achieving higher dressing percentage, and reducing abdominal fat deposition are essential facets of broiler chicken production. Therefore, this study aimed to investigate the effects of Nano-Fe oxide on growth, excreta ammonia emission, carcass criteria, and meat quality of male broiler chicken.

## 2 Materials and methods

### 2.1 Experimental design and dietary treatments

The animal study protocol received approval from the Institutional Animal Care and Use Committee at the University of Alexandria, Egypt (AU08220810298). A total of 192 one-day-old male Ross 308 broiler chicks (with an average body weight of 42.98 ± 0.2 grams) were allocated to three treatment diets, each containing Nano-Fe oxide at 0, 20, or 40 mg/kg, respectively, over a 42-day period. Each treatment group consisted of eight replicates, with eight broiler chicks in each replicate. The birds were fed three different levels of Nano-Fe within a basal control diet: 0, 20, and 40 mg/kg Nano-Fe for the three treatments. The study comprised two phases: the starter phase (0 to 21 days) and the finisher phase (22 to 42 days). Notably, the 20 mg and 40 mg Nano-Fe oxide/kg experimental diets used in this study fell below the minimally recommended dose of 85 mg Fe oxide/kg according to NRC guidelines (17). The chosen Nano-Fe levels aligned with existing research that suggested dietary Fe concentrations ranging from 10 to 60 mg/kg in diets without supplements, up to approximately 160 mg in diets supplemented with 140 mg Fe-Gly or 100 mg Nano-Fe (18, 19). The diets were meticulously crafted to adhere to the precise nutritional requirements outlined for Ross 308 broilers (Table 1), across all growth phases. Throughout the experimental period, the chicks had unrestricted access to feed and water. The study was conducted at the Poultry Center within the Faculty of Agriculture at South Valley University in Qena, Egypt. The chicken cages measured 120 × 70 × 50 cm (length, breadth, and height), with each cage considered as a replicate. Within each cage, there were four nipple drinkers and hanging linear feeders. The nipple line height was adjusted as the birds grew. The chicks were kept on a 23/1 light/dark photoperiod during the whole experimental period. The light intensity was maintained at 35 lux until day 7 and was then reduced to 5–10 lux on day 8 onwards. During the dark period, the light intensity remained below 4 lux according to management requirements outlined for Ross 308 broilers. Indoor conditions included an average ambient temperature of 34.5°C (45% relative humidity) from 1 to 21 days of age, followed by 28.5°C, 40% relative humidity, and a temperature-humidity index (THI) of 29.9 from 22 to 42 days of age. The THI calculation is as follows: THI = db°C − ((0.31–0.31RH) × (db°C − 14.4)), where db is the dry bulb temperature in degrees Celsius and RH is the relative humidity percentage/100. THI values calculated were then classified as follows: 27.8 = no heat stress, 27.8–28.9 = moderate heat stress, 28.9–30.0 = severe heat stress, and >30.0 = extremely severe heat stress (20). The main dietary ingredients for broiler chickens included corn, sorghum, and soybean meal. Sorghum stands out as a valuable feed option for poultry due to its cost-effectiveness and favorable nutrient profile. The diets’ chemical compositions in Table 1 were determined according to the methods of the Association of Official Agricultural Chemists (AOAC) (21). While phytase and carbohydrase enzymes may offer benefits in certain scenarios, their inclusion can also increase feed costs. Therefore, to isolate the effects of Nano-Fe supplementation and minimize potential confounding factors, we did not include these enzymes in the experimental diets.

**Table 1:** The basal diet’s chemical composition (as-fed basis).

Ingredients, %	Diet (1–21 days)	Diet (22–42 days)
Ground corn	27.6	30.0
Ground sorghum	27.6	30.0
Soybean meal (44% CP)	28.5	25.0
Corn gluten (60% CP)	9.50	6.0
Premix vit and min^a^	0.30	0.30
Sunflower oil	3.00	5.52
Dicalcium phosphate	2.00	1.80
Limestone	1.00	1.00
Salt	0.38	0.38
DL-methionine	0.04	—
L-lysine HCl	0.10	—
Total	100	100
Analyzed chemical composition, %		
Dry matter	92.5	92.4
Crude protein	23.3	21.6
Ether extract	5.37	5.75
Crude fiber	2.58	3.78
Ash	6.74	6.18
Ca	1.32	1.284
P	0.70	0.721
Fe	0.024	0.026
Threonine (calculated)	0.91	0.80
Lysine (calculated)	1.15	1.02
Methionine (calculated)	0.45	0.41
GE, MJ/kg (calculated)	18.55	19.18

a Supplied per kg diet, biotin (50 mg), pantothenic acid (10,000 mg), folic acid (1,000 mg), nicotinic acid (30,000 mg), A (1900 IU), K3 (1,000 mg), B1 (1,000 mg), B2 (5,000 mg), B6 (1,500 mg), and B12 (0.046 mg) in addition to D3 (1,300 IU), E (10,000 mg), and BHT (10,000 mg) and includes 60 mg of Mn, 50 mg of Zn, 0.1 mg of Se, 4 mg of Cu, 3 mg of I, and 0.1 mg of Co.

### 2.2 Basil selection, collection, and extract preparation for Nano-Fe oxide synthesis

Fresh basil leaves (*Ocimum basilicum*) were sourced from the farm of the Faculty of Agriculture at South Valley University in Qena, Egypt, where basil was cultivated. The leaves were carefully harvested, cleaned under running water, and rinsed with deionized water. After detaching the leaves from the stems, they were left to air dry at room temperature and subsequently ground into a fine powder. In an Erlenmeyer flask, 100 grams of powdered basil sample were mixed with 1,000 milliliters of double-distilled water while continuously swirling for 15 min to create the extract. Once the extract reached room temperature, it was filtered using Whatman filter paper.

### 2.3 Green synthesis of Fe nanoparticles

**Figure 1:**
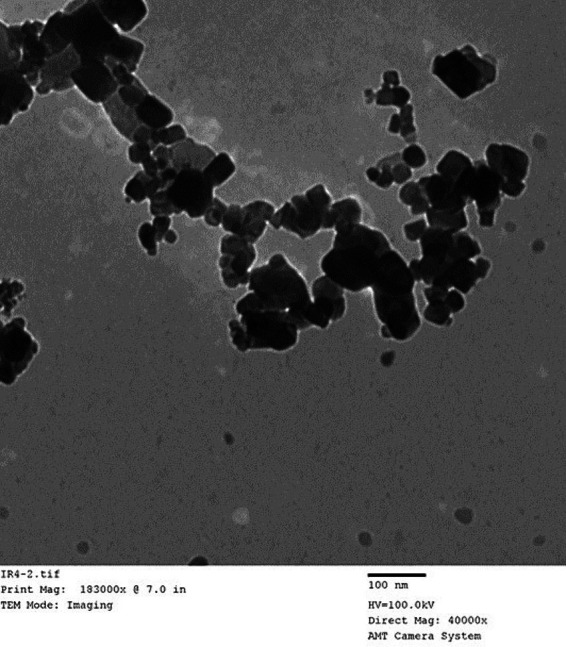
Transmission electron micrographs (TEM) of Nano-Fe.

Following the maceration technique described by Khalil et al. (22), we synthesized green iron oxide nanoparticles using leaf extract from *Ocimum basilicum*. In summary, we heated 30 grams of plant powder and 200 milliliters of distilled water to 80°C for 1 h using a hot-plate magnetic stirrer. Solid residues were removed by triple filtration with Whatman No. 1 filter paper. The resulting filtered solution, with a pH of 5.7, was further heated for 2 h at 85°C. Next, we introduced 100 milliliters of Fe (III) chloride (6 grams) as a precursor salt. The solution transitioned from brownish to violet in color, and its pH was recorded. After allowing the mixture to cool to room temperature, we employed decantation to extract the iron oxide nanoparticles. Following three cycles of distilled water washing, the Fe oxide particles were air-dried at room temperature. These particles were subsequently characterized using a transmission electron microscope (TEM) (Figure 1).

### 2.4 Broiler performance parameters

Throughout the entire experiment, we diligently recorded the body weight of each cage once a week. Additionally, on the days when the birds were weighed, we carefully measured feed residue to determine the amount of feed consumed between weigh-ins. The feed conversion ratio was then calculated by dividing the weight of feed consumed by the body weight gain of each cage.

### 2.5 Ammonia analysis

To assess ammonia excretion, feces were collected at 21 and 42 days of age per cage, following the method proposed by Miles et al. (23). Freshly collected excrement (200 grams) was placed in a 1,000 mL jar. The upper portion of the jar was sealed with a rubber stopper, featuring both an exhaust pipe and an intake pipe connected to a U-shaped bubble absorption tube. Shielded from light, the U-shaped tube contained approximately 10 mL of a 2% boric acid solution. The other end of the U-shaped tube was linked to an inflating pump via a second buffering device, while the intake pipe was connected to a separate buffer device. In an acidic environment, the 2% boric acid solution effectively captured the ammonia gas produced from chicken feces, converting it into stable ${NH}_{4}^{+}$ . Using the Kjeldahl nitrogen determination method, we quantified the nitrogen content in the absorption solution, which was then translated into NH_3_ content based on the unit mass of feces (fresh weight basis).

### 2.6 Carcass criteria and internal organs

A total of 40 birds were selected that represented the pen. Each treatment group consisted of eight replicates, with five broiler chicks in each replicate for measuring the carcass criteria and internal organs at 42 days of age. These birds were weighed, humanely slaughtered using the Halal method, and then plucked. The remaining portion of the body was weighed to determine the dressed weight after removing the head, neck, shanks, viscera, abdominal fat, digestive tract, spleen, liver, gizzard, and heart. The dressing percentage was calculated as the ratio of dressed weight to live weight, multiplied by 100. Additionally, the individual weights of each bird’s liver, heart, empty gizzard, and abdominal fat were recorded, and their proportions relative to the live body weight were determined.

### 2.7 Meat quality measurements

To evaluate water holding capacity (WHC) and cooking loss, we examined the left side of the breast muscle and the left leg in 40 birds per treatment consisted of eight replicates, with five broiler chicks in each replicate. The low-speed centrifugation technique was employed to quantify WHC in breast muscles with minimal adjustments (24). Specifically, 10 grams of intact breast muscle were placed in a falcon tube containing glass beads and centrifuged for 20 min at 10,000 g and 5°C. The resulting precipitated meat was promptly removed, dried using filter paper, and reweighed. WHC was calculated based on the weight loss in muscle samples after centrifugation. Cooking loss was also determined, as previously described (25). In summary, muscle fillets were individually placed in thin-walled, thermotolerant polyethylene bags and cooked in a water bath until their core temperatures reached 70°C. Subsequently, the samples were refrigerated in crushed ice until they reached 5°C, and cooking loss was calculated by reweighing them. Additionally, samples of liver, breast, leg, and blood were collected and stored at −20°C for subsequent iron (Fe) analysis.

### 2.8 Fe analysis

Blood was collected from the wing veins of 40 birds per treatment (5 birds per replication) and placed in vacutainer tubes to obtain serum. Samples of breast, leg, liver, and serum were obtained from slaughtered chickens (*n* = 40) and immediately placed in −20°C freezers for Fe analysis. The Fe concentrations were measured using an atomic absorption spectrophotometer (Perkin Elmer Analyst 800 model, Shelton, CT, United States).

### 2.9 Statistical analysis

In this study, a complete randomized design was employed. The data underwent analysis using SAS 9.2 software, utilizing the General Linear Models (GLM) technique for statistical assessment SAS Institute (26). The supplementation dosage remained constant throughout the model. Growth performance was evaluated at the cage level, while ammonia, carcass criteria, meat quality, and tissue iron content were assessed at the individual bird level. Graphs and a normal distribution test (specifically, the Anderson-Darling test for normality) were generated using GraphPad Prism software, version 9 (GraphPad Software, La Jolla, CA, United States). The effects of increasing Nano-Fe supplementations were determined through orthogonal polynomial contrasts. Duncan multiple range tests were employed for mean comparisons. A significance level of *p* < 0.05 was utilized, with *p*-values less than 0.001 expressed as “<0.001.”

## 3 Results

### 3.1 Broiler performance

The findings demonstrated that Nano-Fe oxide supplementation significantly increased (*p* < 0.05) body weight at starter and finisher periods as well as daily body weight gain compared to control at overall periods (Table 2). In comparison to the control group, the Nano-Fe oxide groups 20 mg/kg and 40 mg/kg improved (*p* < 0.05) body weight and body weight gain under hot climatic conditions. Furthermore, there were no significant differences between 20 mg/ kg and 40 mg/kg in body weight and body weight gain. Adding Nano-Fe oxide to broiler diets, improved (*p* < 0.05) feed conversion ratio throughout the experimental finisher and whole periods (Table 3). Under hot climatic conditions, the supplementation of 40 mg/kg Nano-Fe oxide had a significantly better feed conversion ratio than 20 mg/ kg Nano-Fe oxide and control. The feed intake did not show any significant difference among groups during the experimental periods under hot climatic conditions (Table 3).

**Table 2:** The effects of Nano-Fe oxide on broiler body weight and weight gain.

Items	Body weight, g			Body weight gain, g		
	1 d	21 d	42 d	1–21 d	21–42 d	1–42 d
Nano-Fe levels, mg/kg						
0	42.50	709.50^b^	1939.50^b^	667.00^b^	1230.00^b^	1897.00^b^
20	42.25	747.63^a^	2097.38^a^	705.38^a^	1349.75^a^	2055.88^a^
40	42.13	763.50^a^	2086.88^a^	721.38^a^	1323.38^a^	2044.75^a^
SEM	0.161	13.62	24.33	13.55	27.69	24.64
*p*-value						
ANOVA	0.268	0.030	0.001	0.028	0.009	0.001
Linear	0.115	0.010	0.001	0.009	0.026	0.001
Quadratic	0.755	0.512	0.055	0.507	0.093	0.089

^a,b^Means with various superscripts in a column differ significantly (*p* < 0.05). d, days of age. SEM, the pooled standard error of the means (*n* = 8).

**Table 3:** Effects of Nano-Fe oxide on feed intake and feed conversion ratio of broilers.

	Feed intake, g			Feed conversion ratio		
	1–21 d	21–42 d	1–42 d	1–21 d	21–42 d	1–42 d
Nano-Fe levels, mg/kg						
0	902	2,329	3,231	1.352	1.893^a^	1.703^a^
20	911	2,424	3,334	1.292	1.796^b^	1.622^b^
40	931	2,258	3,189	1.291	1.706^b^	1.560^c^
SEM	16.59	29.14	32.16	0.035	0.069	0.035
*p*-value						
ANOVA	0.450	0.257	0.359	0.325	0.037	0.017
Linear	0.223	0.475	0.687	0.185	0.029	0.005
Quadratic	0.777	0.138	0.173	0.487	0.870	0.824

^a,b^Means with various superscripts in a column differ significantly (*p* < 0.05). d, days of age; SEM, the pooled standard error of the means (*n* = 8).

### 3.2 Ammonia contents

**Figure 2:**
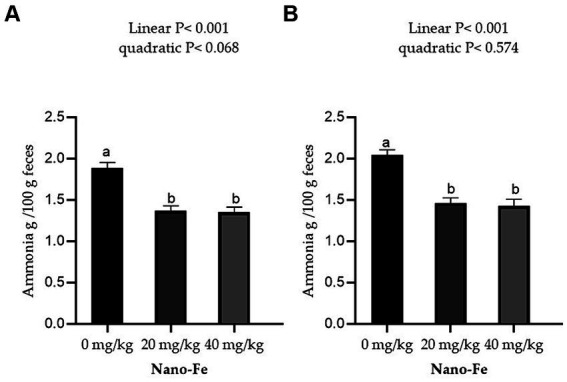
Effects of Nano-Fe oxide supplementation on broiler chicken feces ammonia contents at 21 **(A)** and 42 **(B)** days of age. Bars with different letters (a, b) are significantly different (*p* < 0.05). SEM: Standard error of the means (*n* = 8).

Effects of Nano-Fe oxide supplementations on feces ammonia content during 21 days of age and 42 days of age are presented in Figure 2. The ammonia content of excreta was significantly decreased (*p* < 0.05) with increasing Nano-Fe oxide levels in broiler diets compared to control during 21 and 42 days of age under hot climatic conditions. In addition, there were no significant differences in the ammonia concentration of excreta between 20 and 40 mg/kg.

### 3.3 Carcass criteria

According to the data of carcass criteria, broilers fed diets containing Nano-Fe oxide at 20 mg/kg and 40 mg/kg showed increases (*p* < 0.05) in dressing percentage and decreases (*p* < 0.05) in abdominal fat compared to control (Table 4). However, there were no differences in the dressing percentage and abdominal fat between 20 mg/kg and 40 mg/kg under hot climatic conditions. Supplementation of Nano-Fe oxide had no effect (*p* > 0.05) on the percentages of liver, heart, gizzard, and spleen of broilers.

**Table 4:** Effects of Nano-Fe oxide on dressing, abdominal fat, and internal organs (as the percentages of live body weight) of broiler chickens (42 days of age).

Items	Dressing %	Abdominal fat %	Liver %	Heart %	Gizzard %	Spleen %
Nano-Fe, mg/kg						
0	74.19^a^	0.78^a^	1.89	0.42	1.26	0.10
20	78.27^b^	0.56^b^	1.90	0.44	1.30	0.11
40	78.75^b^	0.53^b^	1.89	0.44	1.30	0.11
SEM	6.19	0.09	0.13	0.02	0.07	0.01
*p*-value						
ANOVA	0.011	0.025	0.460	0.814	0.924	0.828
Linear	0.007	0.020	0.302	0.570	0.718	0.935
Quadratic	0.127	0.641	0.491	0.777	0.874	0.548

^a,b^Means with various superscripts in a column differ significantly (*p* < 0.05). SEM, the pooled standard error of the means (*n* = 8).

### 3.4 Physicochemical characteristics of meat

**Figure 3:**
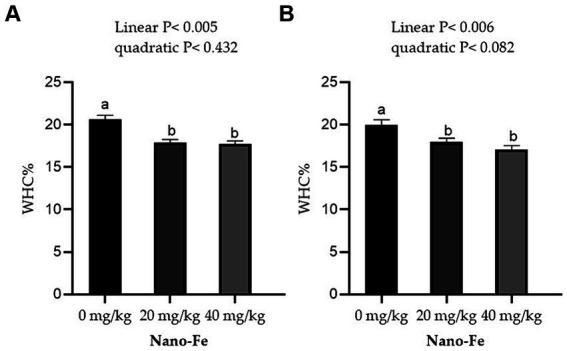
Effects of Nano-Fe oxide supplementation on water holding capacity (WHC) of the breast **(A)** and leg **(B)** muscles in broilers at 42 days of age. Bars with distinct letters (a, b) differ significantly (*p* < 0.05). SEM: Standard error of the means (*n* = 40).

**Figure 4:**
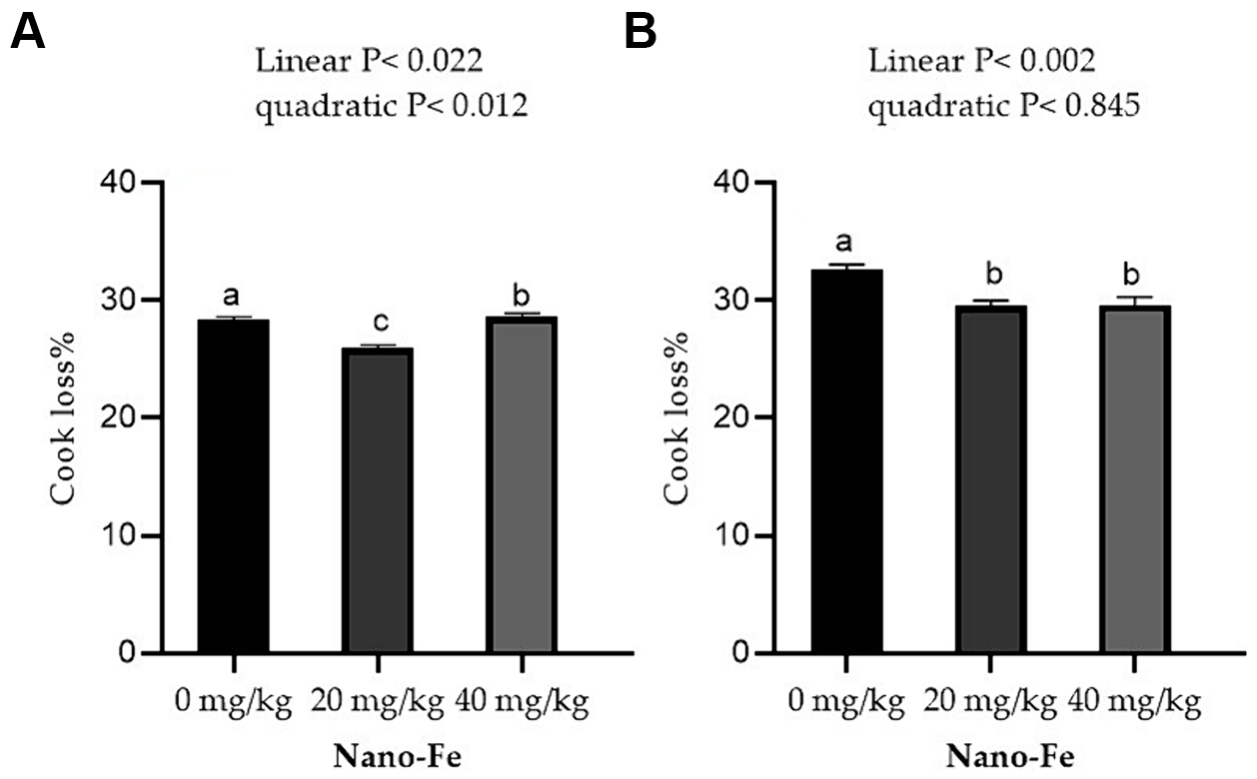
Effects of Nano-Fe oxide supplementation on cooking loss of the breast **(A)** and leg **(B)** muscles in broilers at 42 days of age. Bars with distinct letters (a, b, c) differ significantly (*p* < 0.05). SEM: Standard error of the means (*n* = 40).

Regarding the physicochemical qualities of meat, the WHC % of the breast muscles and leg muscles improved linearly (*p* < 0.05) with respect to the levels of Nano-Fe oxide at 42 days of age in hot climates (Figure 3). Under hot climate conditions, the cook loss percentage of the breast (*p* < 0.05) and leg (*p* < 0.05) muscles at 42 days of age was lowered at 20 mg/kg and 40 mg/kg Nano-Fe oxide levels, respectively (Figure 4). The results revealed that WHC% in breast or leg meat was not different between 20 mg/kg and 40 mg/kg Nano-Fe oxide. However, Nano-Fe oxide supplementation at 20 mg/kg decreased cook loss compared to 40 mg/kg in breast muscles, but no differences were found in leg meat under hot climatic conditions.

### 3.5 Fe content in tissues

**Figure 5:**
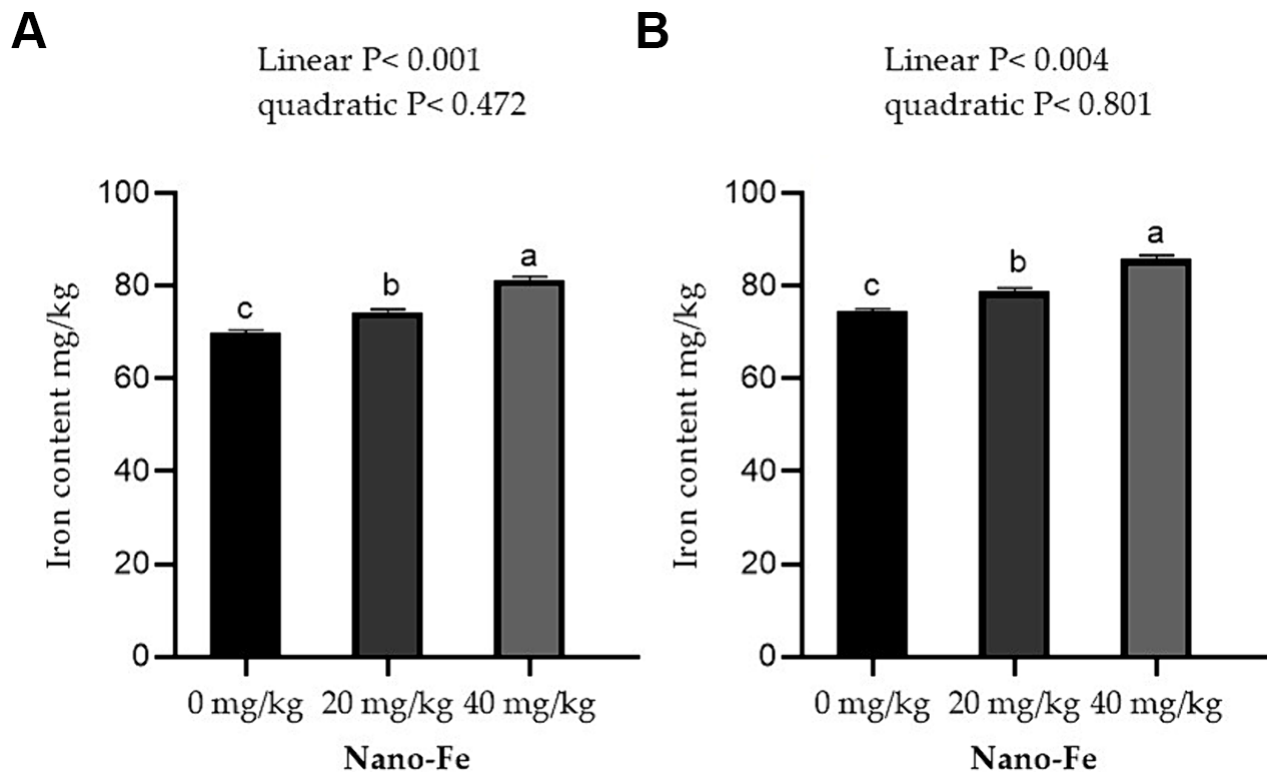
Effects of Nano-Fe oxide supplementation on Fe content in the breast **(A)** and leg **(B)** muscles of broilers at 42 days of age. Bars with different letters (a, b, c) are significantly different (*p* < 0.05). SEM: Standard error of the means (*n* = 40).

**Figure 6:**
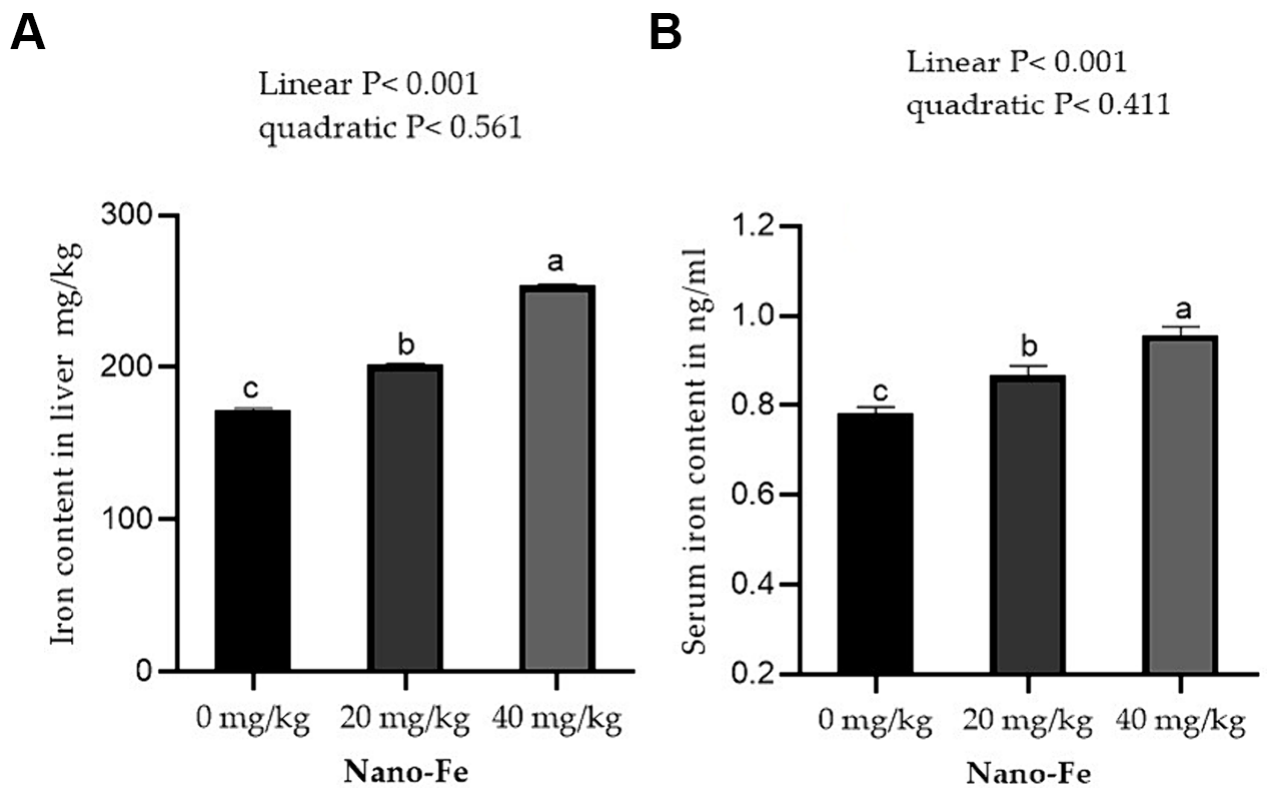
Effects of Nano-Fe oxide supplementation on Fe content of the liver muscles **(A)** and serum **(B)** in broilers at 42 days of age. Bars with different letters (a, b, c) are significantly different (*p* < 0.05). SEM: Standard error of the means (*n* = 40).

Supplementation of Nano-Fe oxide to broiler diets improved (*p* < 0.05) the Fe contents in the breast and leg meat compared to control at 42 days of age (Figure 5). Overall, the Fe content in breast and leg meat was higher in birds fed on Nano-Fe oxide at 40 mg/kg, compared to birds fed on Nano-Fe at 20 mg/kg and control diets, respectively. The Fe content in liver tissues and serum increased (*p* < 0.05) with the increasing levels of Nano-Fe oxide levels compared to control under hot climatic conditions (Figure 6).

## 4 Discussion

In the current study, the iron (Fe) concentrations in the starter and grower diets for broilers were 0.024 and 0.026%, respectively. Additionally, green Nano-Fe was supplemented to the broiler diets at levels of 20 mg/kg and 40 mg/kg. The Fe concentrations in these diets varied, with levels typically ranging from 20 to 80 mg/kg as in other previous studies (17–19). The addition of different iron (Fe) concentrations to broiler diets from previous studies reveals inconsistent outcomes, suggesting a complex interplay of factors influencing Fe utilization and its effects on broiler performance. One significant factor contributing to these inconsistencies is the form and bioavailability of Fe sources used in the diets. Research has shown that the bioavailability of Fe can vary significantly depending on its chemical form and particle size. For example, studies comparing the efficacy of various Fe sources, such as inorganic Fe salts, organic Fe chelates, and Fe nanoparticles, have demonstrated differences in their absorption and utilization by broilers (27, 28). Additionally, variations in dietary composition and nutrient interactions can impact Fe absorption and utilization in broilers. Certain dietary components, such as phytate, can form complexes with Fe, reducing its bioavailability in the gastrointestinal tract. Conversely, other dietary factors, such as vitamin C and organic acids, may enhance Fe absorption (29). Furthermore, genetic factors play a role in determining broiler Fe requirements and response to Fe supplementation. Variations in broiler genetics can influence Fe metabolism and utilization efficiency, affecting growth performance and other physiological parameters (30).

In the current study, supplementation of Nano-Fe oxide at 20 mg/kg and 40 mg/kg to broiler diets improved growth performance compared to control. Green nanotechnology feeding is one of the most potential nutritional techniques, and it deserves more research to improve productive performance of broiler chickens. Fe is a necessary nutrient for chickens, and its bioavailability can be increased by using nanoparticles. Although nanoparticles frequently have more surface area, nanoparticles may be better absorbed in the digestive system. The improved bioavailability of Nano-Fe oxide during hot climatic conditions can benefit broiler growth and overall health. Because Fe is a component of hemoglobin (31), it has a significant role in cellular and whole-body energy metabolism and is essential for protein metabolism (32), chickens are particularly vulnerable to Fe deficiency. Utilizing Nano-Fe in poultry feed displays potential for enhancing feed efficiency. Research on incorporating green Nano-Fe oxide as a feed supplement for poultry in hot climates is limited. Our recent study demonstrates that supplementing Nano-Fe in broiler diets enhances feed conversion ratio between days 21–42 and 1–42 in hot climates, which holds significant importance for the economic viability of poultry production. Few investigations have explored the use of green Nano-Fe as a dietary additive for chickens. Rehman et al. (33) reported that the addition of Nano-Fe and xylanase to broiler feed increased body weight by 45% and improved the feed conversion ratio values at 35 days of age than the control group. The addition of 40–160 mg Fe/kg from Fe-Gly resulted in increasing responses, with the highest body weight gain and feed conversion ratio recorded in broiler chicks fed 100 mg Fe/kg (19). Nikonov et al. (34) observed enhanced production rates in broiler chickens by incorporating Fe oxide nanoparticles into their diet, without any adverse effects. Similarly, Srinivasan et al. (19) discovered that adding 10–20 mg/kg of FeO nanoparticles to *Macrobrachium rosenbergii* feed led to improved growth and satisfactory feed conversion ratios. However, supplementing prawn feed with 30–50 mg/kg FeO nanoparticles resulted in negative effects, increasing feed conversion ratios. Sarlak et al. (35) demonstrated that dietary Fe supplementation in chicken diets improved performance parameters such as feed intake and feed conversion ratio compared to control treatments. Iron is crucial for hemoglobin and myoglobin, facilitating oxygen delivery and cellular utilization. Supplementing Nano-Fe in broiler diets notably increased body weight gain by 8% compared to diets lacking Nano-Fe (36). Furthermore, in the current study, the ammonia content of feces was decreased with increasing green Nano-Fe levels compared to control under hot climatic conditions. Nano-Fe might help to upregulate the functional pathway of nitrogen metabolism in intestinal microbes, increasing the use of nitrogenous substances in the host intestine and lowering ammonia removal through manure (37, 38). Importantly, fulvic acid supplementation to broiler diets decreased the fecal ammonia output in a dose-dependent manner as well as the urease activity (39). The microbial production of uric acid and urea in feces produces a significant amount of ammonia, which causes respiratory illnesses and chronic stress in livestock and poultry (38, 40). Ammonia is created through the deamination of amino acids and the hydrolysis of urea. Changes in ammonia content, as well as ammonia uptake through epithelial cells, have an impact on the microbiota (40). Furthermore, as ammonia levels in the gut dropped, the compensatory effect of ammonia on intestinal cells was reduced, resulting in improvements in the intestinal barrier and histomorphology of the host intestine (41). In the current study, Fe in the breast, leg, liver, and blood was significantly enhanced by adding Nano-Fe oxide to broiler diets. Fe is stored in large quantities in the body, primarily in the liver, and bone marrow reticuloendothelial cells (31). Dietary demands for Fe can be modulated to increase or decrease its rate of absorption via various known pathways depending on the body’s Fe status (3). These processes are linked to receptors on the surface of enterocytes, such as the heme carrier protein 1, which is responsible for heme-Fe absorption in the intestine (42), and the divalent metal transporter 1, which can take inorganic Fe^2+^ and release it directly into the cytoplasm (43). Given that organic Fe absorbs more readily than inorganic Fe, animal byproducts containing muscle tissue and blood have higher Fe availability for poultry (3). Ma et al. (44) investigated the dietary Fe needs of broilers aged 1 to 21 days and discovered that 97 to 136 mg Fe/kg was necessary to sustain their complete expression in various tissues. In addition, serum ferritin levels were considerably higher in diets supplemented with 75, 150, or 300 mg/kg Fe, but not in diets supplemented with 600 mg/kg Fe (45). The Fe content of chick serum increased progressively as the Fe level in the diet increased (35). A large dose of ferrous methionine dramatically raised the hepatic Fe concentration in Ross broilers, according to research by Seo et al. (46). According to Ma et al. (47), the Fe content in broiler liver steadily decreased when dietary Fe levels increased beyond 120 mg/kg. This might be the result of the liver’s ability to keep the right ratio of Fe in the body, preventing excessive Fe deposition that could be harmful. The broiler strains and variety, as well as their feed, may be responsible for the heterogeneity shown in earlier research.

In the current investigation, supplementation of green Nano-Fe oxide to broiler diets significantly improved percentages of carcass dressing and reduced abdominal fat without any side effects on internal organs at 42 days of age under hot climatic conditions. The possible explanation is that Nano-Fe supplementation enhances nutrient absorption and utilization efficiency, leading to more efficient energy metabolism and reduced fat deposition (48). Additionally, Nano-Fe has been reported to exert antioxidant and anti-inflammatory effects, which may contribute to improvements in metabolic health and energy metabolism in broilers (49). Furthermore, Nano-Fe nanoparticles have been shown to interact with cellular signaling pathways involved in lipid metabolism and adipogenesis, potentially leading to alterations in fat deposition patterns (50). The results are consistent with Rehman et al. (33) who found that the Nano-Fe oxide has a lot of potential for usage in chicken feed for large-scale meat production without any negative toxicological effects. Our results are consistent with Lin et al. (51) who reported that weight indices of the liver, kidneys, spleen, thymus, and bursa of Fabricius were not influenced by the different levels of Fe at 50, 70, 90, 110, 130, and 150 mg/kg compared to control. However, it’s important to note that further research is needed to elucidate the specific mechanisms by which dietary Nano-Fe influences energy metabolism and fat deposition in broiler chickens. Future studies should investigate the effects of Nano-Fe supplementation on metabolic parameters, such as hormone levels, enzyme activities, and gene expression related to energy metabolism and lipid metabolism pathways. The current study found that Nano-Fe improved meat quality including WHC and cooking loss under heat stress conditions, which could be attributed to the Fe improving antioxidant activation. Fe has a crucial role in meat quality and Fe metabolism in broilers because it is a necessary component of Fe-containing key enzymes (52). A recent study (53) found that Fe supplementation improved enzymatic antioxidant protection in chicken serum. In addition, Kurtoglu et al. (54) showed that Fe-deficiency anemia lowered plasma antioxidant activities. As far as we are aware, there are no published articles on the effect of green Nano-Fe on broiler chicken carcass criteria and meat quality. Supplementation of Fe to broiler diet with 90 mg/kg Fe decreased drip loss of breast muscle compared to the control diet (47). Fe is a necessary component of hemoglobin in erythrocytes and is used by both hemoglobin and myoglobin (55, 56) for the delivery, storage, and utilization of oxygen in muscles (57). The greatest noticeable indicator of meat quality, color, is mostly determined by hemoglobin and myoglobin (58). Nanotechnology has significant implications for poultry nutrition and it offers targeted nutrient delivery, promotes growth, enhances meat quality, and improves mineral bioavailability. Overall, nanotechnology holds promise for revolutionizing poultry production and addressing industry challenges. The application of Nano-Fe oxide in poultry production holds promise for improving nutritional efficiency and meat quality as well as Fe content in tissue under heat stress. However, thorough research, including safety assessments and regulatory considerations, is necessary to ensure the effective implementation of this technology in the poultry industry. Specific thresholds for Fe levels in broiler chickens for human consumption can differ between countries and regions. For example, in the European Union, the maximum residue limits for Fe in poultry meat is set at 150 mg/kg (Commission Regulation (EU) No 37/2010). In the United States, the Food and Drug Administration (FDA) establishes tolerances for residues of Fe in food products, including poultry meat, based on safety assessments conducted by the agency. However, to provide a more comprehensive assessment of Fe levels in broiler meat and their implications for human consumption, further studies may be warranted. Future research should explore the relationship between dietary Fe supplementation, Fe levels in broiler meat, and potential health outcomes for consumers.

## 5 Conclusion

In conclusion, broiler chickens fed diets containing Nano-Fe oxide at 20 mg/kg and 40 mg/kg exhibited enhanced growth performance, improved meat quality, higher Fe content in tissues, favorable dressing percentage, and reduced abdominal fat. However, no significant effects were observed on internal organs. Future research should explore the impact of green Nano-Fe oxide on additional factors, including the microbiome and gene expression related to immunity and heat stress.

## Data availability statement

The raw data supporting the conclusions of this article will be made available by the authors, without undue reservation.

## Ethics statement

The animal study was approved by Institutional Animal Care and Use Committee of the University of Alexandria, Egypt (AU08220810298). The study was conducted in accordance with the local legislation and institutional requirements.

## Author contributions

YA: Formal analysis, Methodology, Writing – original draft, Writing – review & editing. AE: Conceptualization, Formal analysis, Methodology, Writing – original draft, Writing – review & editing. EE: Conceptualization, Formal analysis, Methodology, Writing – original draft, Writing – review & editing. JL: Writing – original draft, Writing – review & editing. AA-W: Conceptualization, Formal analysis, Methodology, Writing – original draft, Writing – review & editing.
